# Comprehensive Analysis of Transcriptomic Profiles Identified the Prediction of Prognosis and Drug Sensitivity of Aminopeptidase-Like 1 (NPEPL1) for Clear Cell Renal Cell Carcinoma

**DOI:** 10.1155/2023/4732242

**Published:** 2023-02-08

**Authors:** Xiaoyu Wei, Zhongbao Zhou, Guikai Ma, Fengze Sun

**Affiliations:** ^1^Department of Oncology, Tianjin Binhai New Area Hospital of Traditional Chinese Medicine, Tianjin, China; ^2^Department of Urology, Beijing Tiantan Hospital, Capital Medical University, Beijing, China; ^3^Department of Medical Oncology, Weifang People's Hospital, Weifang, Shandong, China; ^4^Department of Urology, Yantai Yuhuangding Hospital, Qingdao University, Yantai, Shandong, China

## Abstract

Aminopeptidase-like 1 (NPEPL1) is a member of the aminopeptidase group that plays a role in the development and progression of various diseases. Expression of NPEPL1 has been reported to be involved in prostate, breast, and colorectal cancers. However, the role and mechanism of NPEPL1 in clear cell renal cell carcinoma (ccRCC) are unclear. The Cancer Genome Atlas (TCGA) and Human Protein Atlas (HPA) databases were used to predict the relationship between clinicopathological features and NPEPL1 expression. Changes in immune status and drug sensitivity with NPEPL1 expression were analyzed by the “CIBERSORT” function in R software. The results found that NPEPL1 expression was upregulated in ccRCC tissues, with expression progressively increasing with ccRCC stage and grade. Patients with high NPEPL1 expression presented with a poor prognosis across different clinicopathological features. Univariate and multivariate Cox regression analyses indicated that aberrant NPEPL1 expression was an independent risk factor for ccRCC. The nomogram showed that NPEPL1 expression improved the accuracy of predicting the prognosis of ccRCC patients. The Gene Ontology (GO) term enrichment analysis and the Kyoto Encyclopedia of Genes and Genomes (KEGG) pathway analysis revealed that NPEPL1 may be involved in the development of ccRCC through the voltage-gated calcium channel complex, channel activity, cAMP signaling pathway, and oxytocin signaling pathway. The coexpression analysis found that NPEPL1 altered tumor characteristics by interacting with related genes. The “CIBERSORT” analysis showed that elevated NPEPL1 expression was followed by an enrichment of regulatory T cells and follicular helper T cells in the microenvironment. The drug sensitivity analysis found patients with high NPEPL1 expression had a higher benefit from axitinib, cisplatin, and GSK429286A. In conclusion, upregulation of NPEPL1 expression was involved in ccRCC prognosis and treatment. NPEPL1 could be used as a therapeutic target to guide clinical dosing.

## 1. Introduction

Renal cell carcinoma (RCC) is one of the most common malignancies among urological carcinomas, representing 80% of renal malignancies [1]. The main pathological types include clear cell RCC (ccRCC), collecting duct RCC, chromophobe RCC, and papillary RCC [2, 3]. ccRCC, as the most common subtype, accounts for approximately 70% of all RCC [4]. Patients are generally found by examination and diagnosed at an advanced stage, with a 5-year survival rate of about 11.7% due to a lack of specific inspection methods [5]. Patients treated with conventional chemoradiotherapy always had poor outcomes. For targeted therapy, some patients may have drug resistance, resulting in poor long-term prognosis, which poses a new challenge for the treatment of renal cancer [6]. With the development of tumor therapy, immune therapy, including immune checkpoint inhibitors (ICIs), has been the most promising choice for ccRCC. The therapeutic mechanism of ICIs is briefly considered to be blocking the abnormal pathways that maintain immune self-tolerance to prevent immune escape. Since antibody-mediated programmed cell death protein 1 (PD-1) blockade was approved by the American Food and Drug Administration (FDA), ICIs have emerged as the new first- and second-line standard of care for patients with intermediate to advanced disease as monotherapy or combination therapy [7, 8]. Despite these therapies being widely used in clinical practice, most RCC patients do not derive lasting benefit from ICI treatment. Thus, understanding the pathogenesis associated with progression and finding new therapeutic markers are important for predicting outcomes and prognosis.

Exogenous amino acids are essential for the survival of tumor cells. The stable state of amino acids and proteins essential for cells depends on the catalytic cleavage of amino acids at the amino terminus of proteins by aminopeptidases [9]. Clinical studies have also shown that cancer patients with high aminopeptidase expression tend to have a poor prognosis [10, 11]. Proliferating active tumor cells may be inhibited by low expression of aminopeptidase. This provides the rationale that aminopeptidase can be used as a new therapeutic approach [12, 13].

Aminopeptidase-like 1 (NPEPL1), a member of the aminopeptidase family, has functions such as manganese ion binding and metalloexopeptidase activity. It plays a role in proteolysis and also takes part in the development and progression of various diseases. NPEPL1 has been reported as one of the prognostic markers of destructive resistance prostate cancer and appeared to be useful in predicting the recurrence-free survival of patients [14]. It can bind directly to miR-19a and take part in the development and progression of breast and colorectal cancers [15–17]. Abnormalities in NPEPL1 may also be closely associated with the development of Alzheimer's disease [18]. Moreover, elevated expression of NPEPL1 and adjacent STX16 could promote the probability of gastrointestinal tumorigenesis [19]. Long-range deletion spanning NPEPL1 and adjacent STX16 is related to rare pseudohypoparathyroidism [20]. However, the relation between NPEPL1 and ccRCC in terms of prognosis and treatments has not yet been completely elucidated.

In this study, we assessed the relationship between NPEPL1 expression and the clinical characteristics of ccRCC patients using the TCGA and Human Protein Atlas (HPA) databases. We found that high expression of NPEPL1 suggested a poor prognosis for patients. The “CIBERSORT” analysis was then used to validate the correlation between NPEPL1 expression and immune status. We found that NPEPL1 might affect a variety of immune cells. Finally, we also predicted drug sensitivity in patients with high NPEPL1 expression, who were especially sensitive to cisplatin, axitinib, and GSK429286A. Therefore, upregulation of NPEPL1 expression was involved in ccRCC prognosis and treatment and guided the application of therapeutic drugs.

## 2. Method

### 2.1. The Expression of NPEPL1 in TCGA and HPA Databases

This study was conducted according to the method of Dr. Zhou et al. [21]. The TCGA database was used to collect clinical data (containing 539 KIRC cases), including gender, age, grade, TNM stage, pathological stage, survival status, and survival time. Protein expression of NPEPL1 in renal tissue and KIRC was obtained from the HPA database.

### 2.2. Survival Analysis

The R package “survival” was used to analyze survival data. Patients were graded into high and low expression groups according to the median value set for NPEPL1 expression in the tumor. The relationship between NPEPL1 expression and clinical outcomes was detected.

### 2.3. Univariate and Multivariate Logistic Regression Analyses

The association between NPEPL1 expression and clinicopathological characteristics and overall survival (OS) can be assessed using univariate Cox regression. Multivariate Cox regression clarified the importance of NPEPL1 in the survival of ccRCC patients. When the *P* value was less than 0.05, we considered that the factor showed significance in the OS of the patients.

### 2.4. Evaluation and Construction of Prognostic Nomogram

We drew a prognostic nomogram to visually show the prognostic predictors of ccRCC patients (age, T, N, M, histological grade, and NPEPL1 expression level) on OS. The reliability and accuracy of the nomogram were evaluated by the calibration curve.

### 2.5. Analysis of Differentially Expressed Genes and Their Functions

Differential expression genes (DEGs) were analyzed by the R package “limma” between high and low NPEPL1 expression. The false discovery rate (FDR) was performed to correct *P* value for multiple test correction. When |log2FC| value was set at more than 1 and FDR less than 0.05, DEGs were selected and included in the Gene Ontology (GO) term enrichment analysis and the Kyoto Encyclopedia of Genes and Genomes (KEGG) pathway analysis.

### 2.6. Immune Landscape Assessment

To describe the link between the immune microenvironment and NPEPL1 expression, “CIBERSORT” analysis was used to collect data on immune cell infiltration in ccRCC patients and was evaluated by R software. “Spearman” analysis was used to clarify the correlation between NPEPL1 and the immune microenvironment in tumor development. Comparison of differentially expressed immune checkpoints between NPEPL1 high and low expressing groups was performed to clarify immune mechanisms by which NPEPL1 mediates tumorigenesis development.

### 2.7. Sensitivity to Drugs of NPEPL1

The R package “pRRophetic” was employed to identify the half-maximal inhibitory concentrations (IC_50_s) of commonly used drugs, including cisplatin, axitinib, ICIs, and others, in order to estimate the sensitivity of high and low NPEPL1 expression to different drugs. The difference in IC_50_ values between high- and low-expression groups was estimated by the Wilcoxon signed rank test.

### 2.8. Statistics Analysis

All statistical analyses were calculated using R software (version 4.0.2). The Kaplan–Meier analysis was used to assess the impact of NPEPL1 on patients' survival. Univariate Cox regression was performed to evaluate the relationship between clinicopathological characteristics and OS, and multivariate Cox regression was used to clarify that NPEPL1 was an important factor for patients' survival. The Wilcoxon rank-sum test was used to evaluate the relation between NPEPL1 and molecular functions. The results were deemed statistically significant when the *P* value was less than 0.05.

## 3. Result

### 3.1. NPEPL1 Expression in Pan-Cancer Analysis

**Figure 1:**
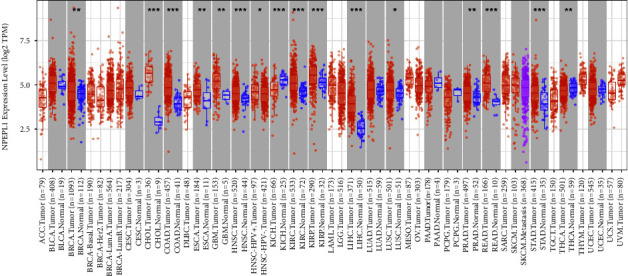
Pan-cancer-related expression pattern of NPEPL1. (^*∗*^: *P* < 0.05, ^*∗∗*^: *P* < 0.01, ^*∗∗∗*^: *P* < 0.0001).

NPEPL1 expression was detected in 32 cancers, as shown in Figure 1. Compared to normal tissues, NPEPL1 expression was higher in 13 types of cancer, including KIRC, and lower in thyroid carcinoma and kidney chromophobe. The data suggested that NPEPL1 was differentially expressed in different tissues and in different types of cancer in the same tissue.

### 3.2. The Expression Characteristics of NPEPL1 in KIRC

**Figure 2:**
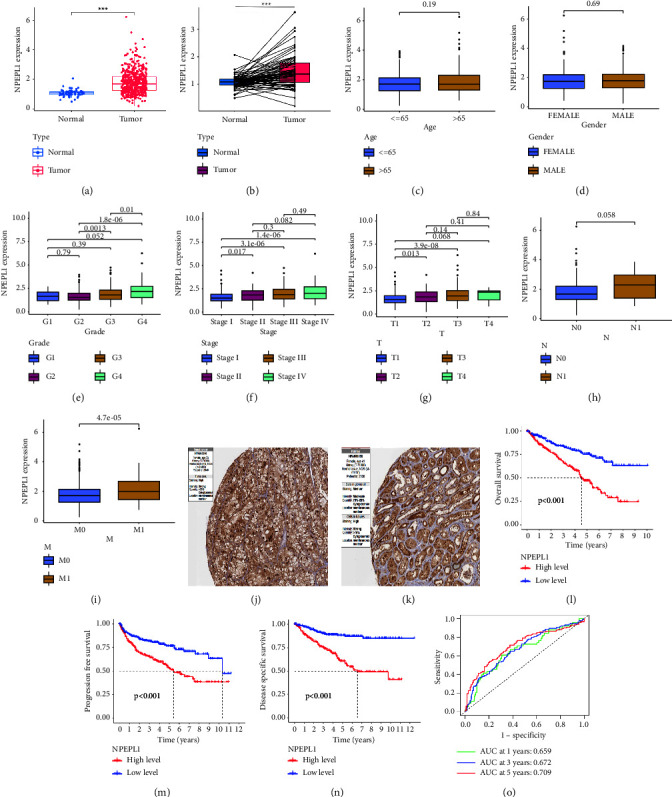
The expression of NPEPL1 and related clinical features in ccRCC. The NPEPL1 expression between normal and tumor tissues. (a, b) The NPEPL1 expression according to different clinical features, including age (c); gender (d); grade (e); stage in stages I II, III, and IV (f); T stage in T1, T2, and T3 (g); N stage in N0 and N1 (h); M stage in M0 and M1. (i) The protein expression of NPEPL1 between normal and tumor tissue. (j, k) The overall survival, progression-free survival, and disease-specific survival between low and high NPEPL1 expression. (l, m, n) AUC curve related to OS (o).

The patients were divided into various groups according to their clinicopathological features, including age (less than 65 years old and more than 65 years old), gender (male and female), grade (grade 1, grade 2, grade 3, and grade 4), stage (stage I, stage II, stage III, and stage IV), and TNM stages (T1, T2, T3, T4, N0, N1, M0, and M1). The expression of NPEPL1 in different features was detected to clarify its role in ccRCC, in which the expression was higher in tumor tissues (Figures 2(a) and 2(b)). The gender and age of tumor patients were not affected by expression (Figures 2(c) and 2(d)). With the increase in tumor stage and grade, the expression level of NPEPL1 increased significantly (Figures 2(e)–2(i)). The expression affected the metastasis of the tumor, rather than lymph node metastasis. This result identified that the high NPEPL1 expression was related to the advanced stage of ccRCC. The HPA database was also applied to suggest that NPEPL1 protein overexpression was correlated with the development and progression of ccRCC (Figures 2(j) and 2(k)).

### 3.3. Relationship between NPEPL1 Expression and ccRCC Prognosis

**Figure 3:**
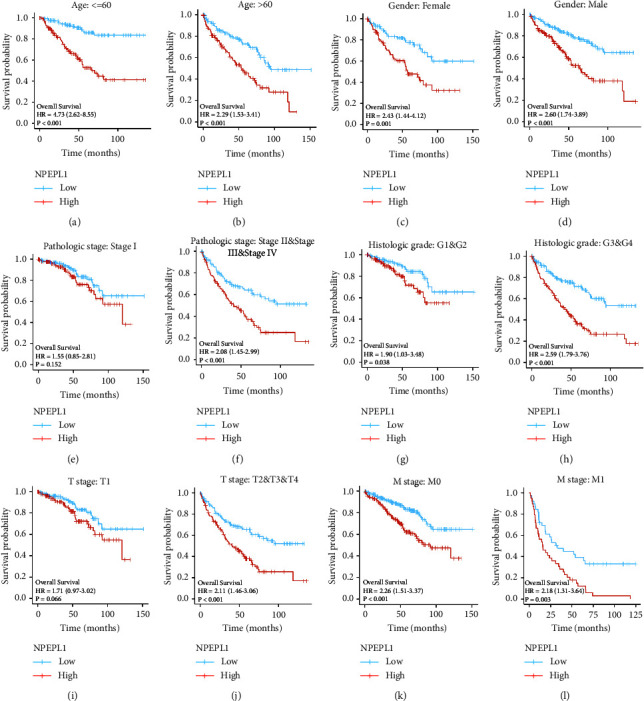
The OS between low and high NPEPL1 expressions according to clinicopathological features, including age between lower than 60 years old and higher than 60 years old (a, b); gender between male and female (c, d); stage between stage I and stage II, III, and IV (e, f); grade between grade 1 and 2 and grade 3 and 4 (g, h); T stage between T1 and T2, 3 and 4 (i, j); M stage between M0 and M1 (k, l).

We classified the 539 patients in the TCGA-KIRC cohort into high and low NPEPL1 groups according to the median expression of NPEPL1 in tumor tissue as the cutoff. The details of the patients are shown in Table 1. The significant difference was presented in OS (*P* < 0.001), progression-free survival (PFS, *P* < 0.001), and disease-specific survival (DSS, *P* < 0.001) (Figures 2(l)–2(n)). The area under the curve (AUC) at 1 year, 3 years, and 5 years were 0.659, 0.672, and 0.709, respectively, which were better than 0.6, implying good predictive value (Figure 2(o)). Next, the correlation between survival and NPEPL1 expression was performed according to subgroups of clinicopathological features. The high expression of NPEPL1 indicated poor survival in clinical features including age (less than 60 years old and more than 60 years old) and gender (male and female) (Figures 3(a)–3(d)). For the pathologic stage, the patients with high NPEPL1 expression presented poorer outcomes in stages II, III, and IV (*P* < 0.001), whereas the difference was not significant in stage I (*P*=0.152). (Figures 3(e) and 3(f)). For the histologic grade, the high NPEPL1 expression meant worse survival in both grades I and II (*P*=0.038) and grades III and IV (*P* < 0.001). (Figures 3(g) and 3(h)). The NPEPL1 expression was not correlated with survival in early T stage (*P*=0.066); however, the high NPEPL1 expression implied worse survival in T2, T3, and T4 (*P* < 0.001) (Figures 3(i) and 3(j)). Whether distant metastasis occurred or not, high NPEPL1 expression indicated poor survival (Figures 3(k) and 3(l)). These results indicate that the higher NPEPL1 expression meant poor prognosis for ccRCC patients in different clinical features.

### 3.4. Construction and Evaluation of Nomogram

**Figure 4:**
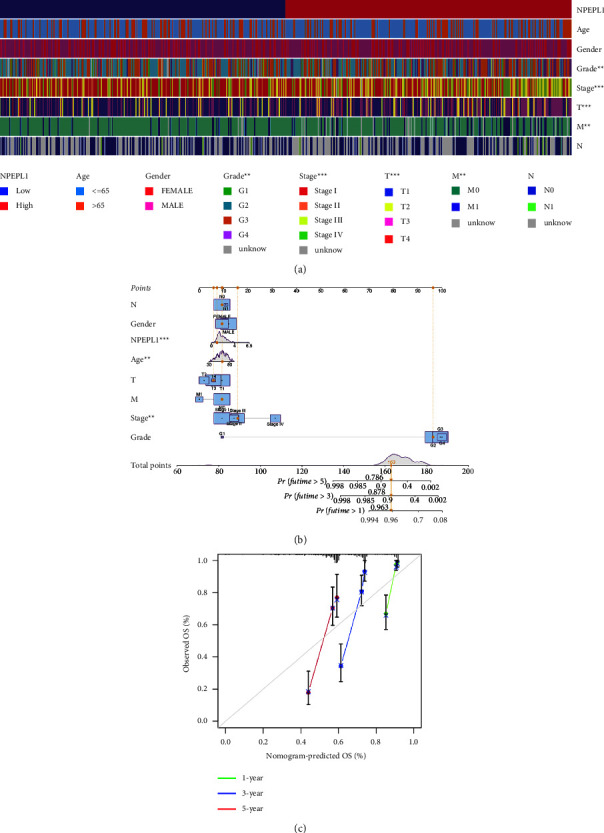
(a) Heatmap showed a significant histologic grade, pathologic stage, and T and M stage between high- and low-expression NPEPL1. (b) Nomogram predicting the probability of patients with OS at 1, 3, and 5 years. (c) The calibration curve shows the actual and predicted survival rates.

The univariate and multivariate analyses identified that M stage, age, and NPEPL1 expression were all independent risk factors for the prognosis of ccRCC (Table 2). Furthermore, the bar plot and table presented that T stage (*P* < 0.001), M stage (*P* < 0.01), pathologic stage (*P* < 0.001), and histologic grade (*P* < 0.01) were notably associated with NPEPL1 expression (Figure 4(a) and Table 3). NPEPL1 expression and clinicopathological features were used to build a nomogram to predict OS at 1, 3, and 5 years in ccRCC patients (Figure 4(b)). High expression of NPEPL1 predicted a poor prognosis. Calibration curves showed the predictive value of the nomogram was consistent with actual results, which demonstrated that the nomogram was robust and precise (Figure 4(c)).

### 3.5. DEGs and Enrichment Analysis of Low and High NPEPL1 Expression

**Figure 5:**
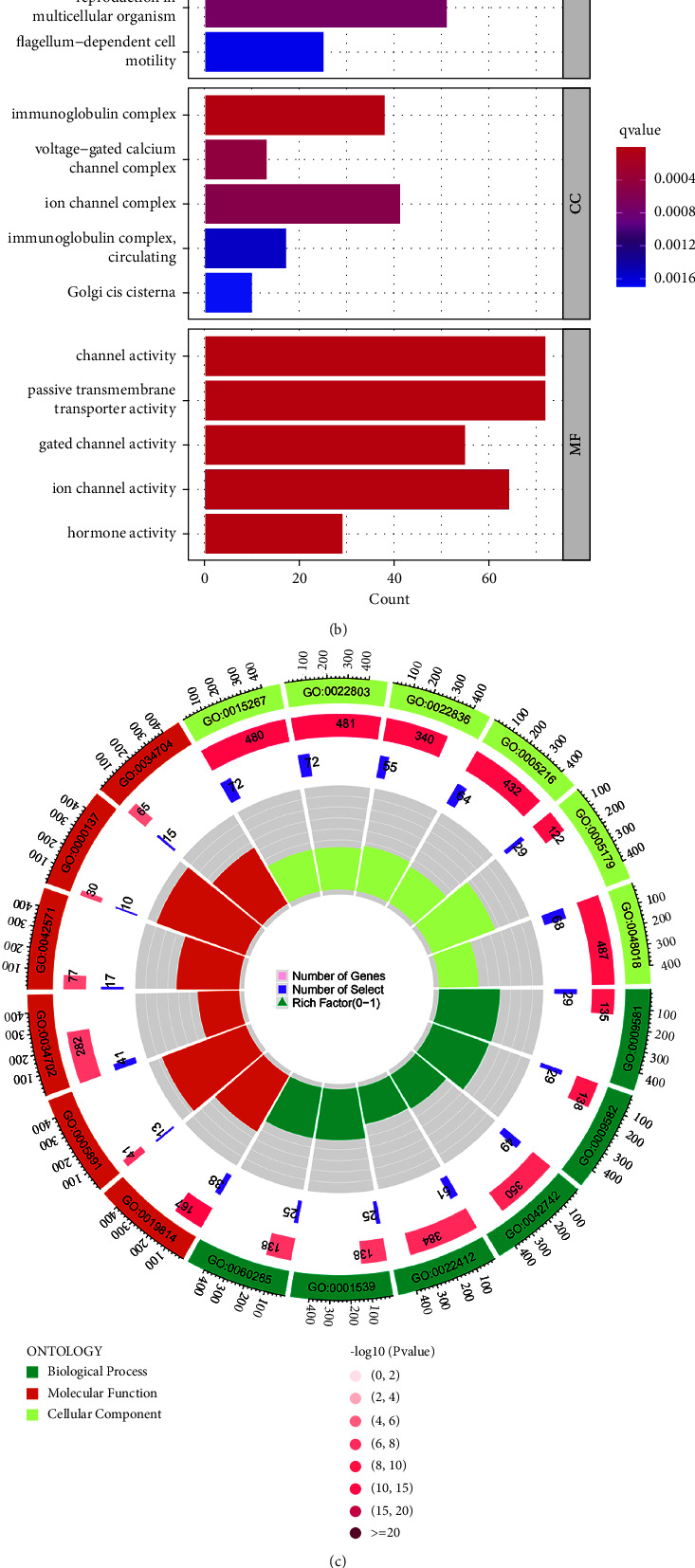
(a) Heatmap of differential expression genes between high- and low-expression NPEPL1. (b, c) Enrichment of DEG for biological processes (BP), cellular components (CC), and molecular functions (MF). (d) KEGG enrichment pathway of DEGs.

Finally, about 5,679 DEGs were determined, of which 5,635 genes were upregulated and 44 genes were downregulated. The top 50 DEGs were mapped by heatmap in Figure 5(a). The GO analysis was used to predict the enrichment analysis of low and high NPEPL1 expression by applied biological process (BP), molecular function (MF), and cellular component (CC) groups. The main enrichment items were detection of external stimulus, detection of abiotic stimulus, immunoglobulin complex, voltage-gated calcium channel complex, channel activity, and passive transmembrane transporter activity (Figures 5(b) and 5(c)). The main KEGG enrichment pathways were neuroactive ligand-receptor interaction, pancreatic secretion, the cAMP signaling pathway, and the oxytocin signaling pathway (Figure 5(d)).

### 3.6. Coexpression Network Construction

**Figure 6:**
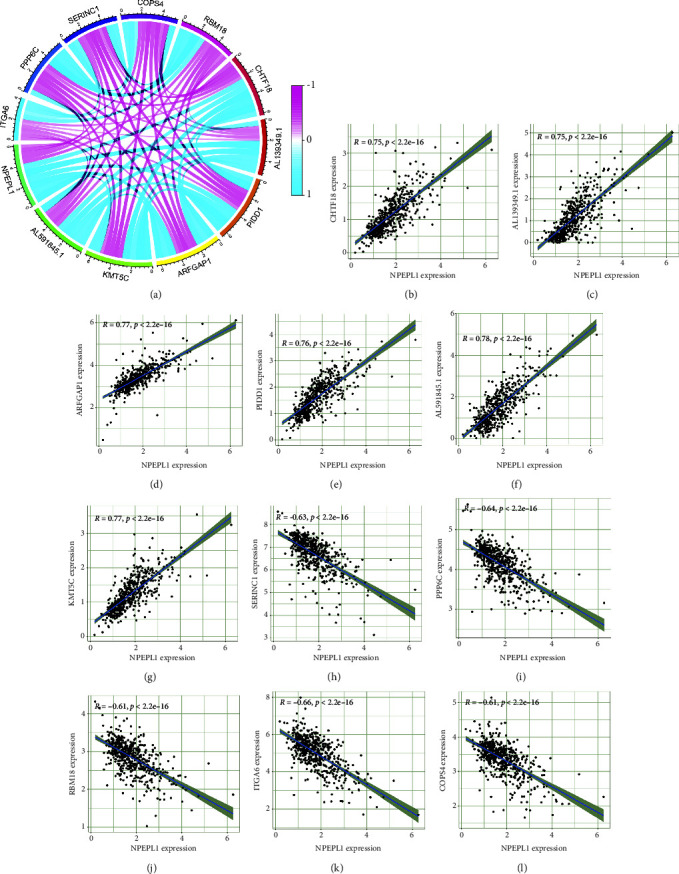
(a) Correlation analysis of NPEPL1 expression. The association of NPEPL1 with the top eleven core genes includes CHTF18 (b), AL139349.1 (c), ARFGAP1 (d), PIDD1 (e), AL591845.1 (f), KMT5C (g), SERINC1 (h), PPP6C (i), RBM18 (j), ITGA6 (k), and COPS4 (l).

The DEGs that interacted directly with NPEPL1 were selected to draw an interaction network using the “limma” R package. The top 11 genes interacted with NPEPL1 closely were performed, including CHTF18, AL139349.1, ARFGAP1, PIDD1, AL591845.1, KMT5C, SERINC1, PPP6C, RBM18, ITGA6, and COPS4 (Figure 6(a)). The NPEPL1 presented high coexpression relationship with CHTF18 (*R* = 0.75), AL139349.1 (*R* = 0.75), ARFGAP1 (*R* = 0.77), PIDD1 (*R* = 0.76), AL591845.1 (*R* = 0.78), KMT5C (*R* = 0.77), SERINC1 (*R* = −0.63), PPP6C (*R* = −0.64), RBM18 (*R* = −0.61), ITGA6 (*R* = −0.66), and COPS4 (*R* = −0.61).

### 3.7. Relation between NPEPL1 and Infiltrating Immune Cells

**Figure 7:**
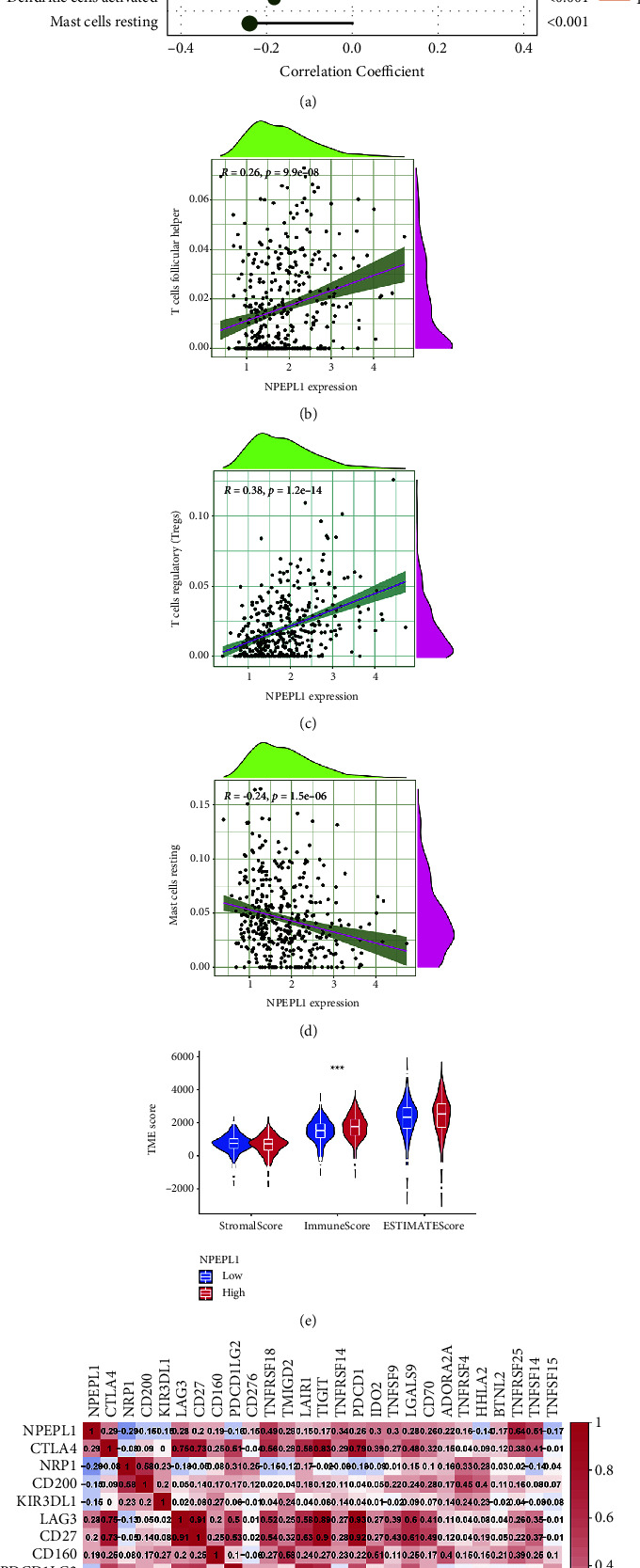
(a) Forest plot of NPEPL1 expression correlation with 24 immune cells. The scatter plot of the correction between NPEPL1 expression and immune cell regulation includes regulatory T cells (b), follicular helper T cells (c), and resting mast cells (d). The pink line in each scatter plot is a fitting linear model, suggesting a significant correlation between immune cells and NPEPL1 expression. (e) The immune microenvironment between high and low expression of NPEPL1, including stromal score, immune score, and ESTIMATE score. (f) Heatmap of immune checkpoints related with NPEPL1.

The occurrence and development of tumor were closely linked to immune cell infiltration. We analyzed the difference of immune cell infiltration between high and low NPEPL1 expression groups, and some infiltrating immune cell subtypes presented significant correlation with NPEPL1, including regulatory T cell, follicular helper T cell, memory B cell, CD8 T cell, activated NK cell, plasma cell, M0 macrophage, CD4 memory resting T cell, monocytes, gamma delta T cell, naïve B cell, eosinophiles, M2 macrophage, resting dendritic cell, activated dendritic cell, and resting mast cell (Figure 7(a)). By analyzing three immune cell subtypes with obvious differences, it was found that regulatory T cells and follicular helper T cells were significantly positively associated with the expression of NPEPL1, while resting mast cells were associated with a significant negative correlation with NPEPL1 expression (Figures 7(b)–7(d)).

### 3.8. Immune Microenvironment and Checkpoints Related with NPEPL1

The analysis of the immune microenvironment identified that high NPEPL1 expression correlated with a high immune score in violin plots, which implied that NPEPL1 could increase immune activity rather than stromal activity to promote the progression of ccRCC (Figure 7(e)). Furthermore, the immune checkpoints related to NPEPL1 were also drawn in a heatmap, in which red meant positive correlation and blue meant negative correlation (Figure 7(f)). The TNFRSF25 and TNFSF14 presented a positive correlation with NPEPL1, while the NRP1 and TNFSF15 had a negative correlation with NPEPL1. These results identified that high NPEPL1 may affect the progression of ccRCC by changing the immune microenvironment.

### 3.9. Drugs Sensitivity of NPEPL1

**Figure 8:**
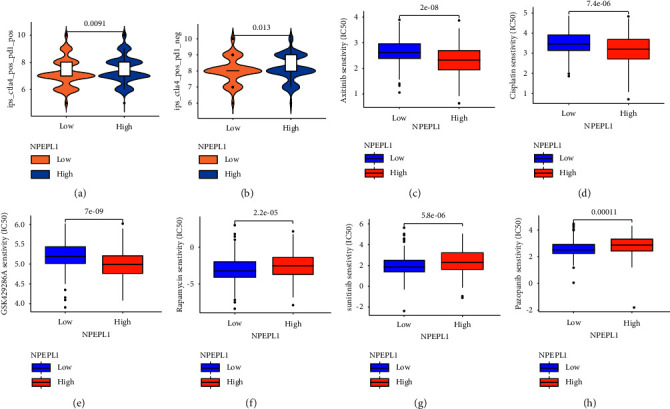
Analysis of drugs susceptibility. Sensitivity to immunotherapy (a, b), axitinib (c), cisplatin (d), GSK429286A (e), rapamycin (f), sunitinib (g), and pazopanib (h).

Checkpoint inhibitors monotherapy and combination therapy with target drugs, and chemotherapy have been the main therapy methods for ccRCC. We also tried to predict whether the NPEPL1 expression was related to sensitivity of ccRCC patients to checkpoint inhibitors, chemotherapeutic agents and common targeted drugs. We found that two groups had a significant difference in response to ctla-4_pos_pd1_neg and ctla-4_pos_pd1_pos, which powerfully predicted that patients with different NPEPL1 expression had a significantly different immunotherapy response (Figures 8(a) and 8(b)). Patients with high NPEPL1 expression had lower IC50 for axitinib (*P* < 0.001, Figure 8(c)), cisplatin (*P* < 0.0001, Figure 8(d)), and GSK429286A (*P* < 0.001, Figure 8(e)), which implied that patients were more sensitive to these drugs. However, the patients with high NPEPL1 expression were not sensitive for rapamycin, sunitinib, and pazopanib, whose IC_50_ was lower in low NPEPL1 expression (Figures 8(f)–8(h)).

## 4. Discussion

Clear cell RCC is the common type of RCC, which is highly malignant with poor prognosis and remains difficult to predict and treat. Monotherapy or combination therapy based on immunotherapy has become the standard treatment strategy for ccRCC, and patients with similar clinical features and the same treatment may have different prognoses [22–24]. Individualized treatment approaches based on the patient's characteristics are important in improving the patient's prognosis. Therefore, it is essential to look for relevant markers to predict prognosis and clarify clinical outcomes after systematic treatment.

The mRNA NPEPL1 is located on chromosome 20q13.32 and encodes probable aminopeptidase-1, whose main function includes manganese ion binding and metalloexopeptidase activity. In the previous study, NPEPL1 had functions in the development and progression of prostate cancer and breast cancer [14–17]. Moreover, NPEPL1 is adjacent to STX16, and the transcript STX16-NPEPL1 is allowed to emerge. The read-through transcript is related to gastrointestinal tumorigenesis and rare pseudohypoparathyroidism [19, 20]. However, the functions of NPEPL1 in the prognosis and treatment of ccRCC were not clear. This study sought to elucidate the character of NPRPL1 in ccRCC.

First, we found that mRNA NPEPL1 was differentially expressed between normal tissues and tumor tissues in different organs via pan-cancer analysis. We also used the TCGA database to analyze the relation between NPEPL1 expression and the clinicopathological features of ccRCC. NPEPL1 expression was higher in ccRCC tissues, and the expression increased gradually with the increase in tumor grade and stage. The HPA database also confirmed that the protein of NPEPL1 was more detectable in tumor tissue. Kaplan–Meier curves were applied to predict the prognosis of ccRCC patients between low and high NPEPL1 expression groups and indicated that the high NPEPL1 expression group had a poor prognosis. The multivariate logistic regression analysis indicated that high NPEPL1 expression was an independent prognostic factor.

Next, the GO analysis was mostly abundant in “detection of external stimulus,” “voltage-gated calcium channel complex,” “ion channel complex,” and “channel activity.” The abnormal activity of channels in a cell member may cause the occurrence of renal cell carcinoma, especially in calcium channels and transient receptor potential (TRP) channels [25–27]. The calcium channel and TRP channels activity broke the balance of proangiogenic and antiangiogenic factors, which could shift towards proangiogenic function [28]. The calcium entry across the plasma membrane accelerated the angiogenesis process by stimulating mature ECs, and TRP channels provided the pathway for the calcium entry signal. The related channel activity also played important roles in drug resistance resisting cell death, tumor stem cell differentiation, tumor microenvironment alteration, and tumors evading immune destruction [29–32]. The blocks of calcium channels and TRP channels were used to decrease occurrence risk of RCC, relieve drug resistance, and improve patient prognosis [33, 34]. Moreover, KEGG analysis was mainly concentrated on protein digestion and absorption, the cAMP signaling pathway, the calcium signaling pathway, and the Ras signaling pathway. The abnormal function of protein digestion and absorption following NPEPL1 expression dysregulation promoted invasion, migration, and drug resistance in ccRCC [35, 36]. With the in-depth understanding of the mechanism of ccRCC development, cAMP and the Ras signaling pathway played a crucial role in regulating biological behaviors [37, 38]. Regulation of some crucial signaling pathways could modulate the growth, invasion, migration, and drug resistance of tumor, become a new target of treatment, and improve the prognosis of tumor patients [39–41].

The eleven proteins coexpressed with NPEPL1 were identified, with six proteins upregulated and five proteins downregulated with the increase in NPEPL1 expression. Among them, PIDD1 has proved to play a positive role with an increase in stage in RCC patients [42]. The expression of SERINC1 exerted a protective effect in the progression of RCC, and ITGA6 expression may be a main factor in the treatment of drug-resistant RCC with valproic acid and interferon-alpha [43, 44]. Although CHTF18 and KMT5C have not been shown to correlate with RCC, they played a role in the development of other tumors; abnormalities in CHTF18 promoted endometrial carcinoma, and KMT5C played a role in non-small cell lung cancer [45, 46].

Tumor immune cell infiltration has been approved to be associated with the prognosis of ccRCC and the response to immunotherapy [47, 48]. The importance of some infiltrating immune cells has been confirmed, including regulatory T cells, CD8 T cells, NK cells, and resting mast cells [49–52]. The immune cell infiltration analysis between high and low NPEPL1 expression also revealed differences in immune cells similar to previous studies. Interestingly, the infiltration level of CD8T cell was high in patients with high NPEPL1 expression, and CD8T cells were a kind of antitumor immune cell [53]. Regulatory T cells, which have a negative effect on antitumor activity, had a higher infiltration level in high NPEPL1 expression patients [54]. These results showed that the immune regulation in tumor tissues was multidirectional, and the antitumor effect was offset by a stronger immunosuppressive environment in patients with high expression of NPEPL1. Moreover, immune checkpoints (TNFRSF25 and TNFSF14) were positively correlated with NPEPL1, which was a prognostic factor of ccRCC and had been confirmed by previous studies [55, 56]. TNFRSF25 could increase the proliferation of regulatory T cells [57–59]. TNFSF15, as the ligand of TNFRSF25, presented a negative correlation with NPEPL1 and played a negative role in regulatory T cells' suppressive ability [57–60]. The inhibitory ability of regulatory T cells was promoted by suppression of TNFSF15 and TNFRSF25 expression. Above all, NPEPL1 expression regulated the distribution of immune cells in tumor tissues through immune checkpoints, which affected the occurrence and development of ccRCC.

ICIs have been proven to play a significant effect in solid tumors, and the activation of tumor immune microenvironment can improve the outcome of ICIs treatment. We found that low and high NPEPL1 expression groups had a significant difference in response to ctla-4_pos_pd1_neg and ctla-4_pos_pd1_pos, which powerfully predicted that patients with different NPEPL1 expression had a significantly different immunotherapy response. Besides, the low NPEPL1 expression group was more sensitive to rapamycin, sunitinib, and pazopanib; the high NPEPL1 expression group was more sensitive to axitinib, cisplatin, and GSK429286A. Axitinib, sunitinib, and pazopanib were all ATP-competitive inhibitors of vascular endothelial growth factor receptors (VEGFRs), which were approved to treat RCC by the FDA [61]. The high NPEPL1 expression group was more sensitive to axitinib, since axitinib was more selective for VEGFRs but not PDGFRs, B-Raf, c-Kit, or Flt-3 [62, 63].

All in all, NPEPL1 expression was upregulated in ccRCC tissues compared to normal tissues and increased with the development and progression of ccRCC. The high NPEPL1 expression was related to poor prognosis and immune responses. Some potential limitations were not ignored in our study. First, more clinical samples were required to confirm that NPEPL1 was an important prognostic factor in ccRCC. Second, the mechanism of NPEPL1 in the development and progression of ccRCC was necessary to identify. Third, the interaction between NREPL1 expression and immune cell infiltration needs to be confirmed by more studies.

## 5. Conclusion

We confirmed the prognostic value of high NPEPL1 expression in ccRCC, which was upregulated with development and progression. NPEPL1 expression plays certain roles in metastasis, metabolism, and the immune microenvironment in ccRCC. We also predicted that patients with high NPEPL1 expression would be more sensitive to some common drugs, including axitinib, cisplatin, and GSK429286A. NPEPL1 could be regarded as a prognostic predictor and therapeutic target in ccRCC patients and guide clinical medication.

## Figures and Tables

**Table 1:** Association between NPEPL1 expression and various clinicopathological characteristics in the TCGA database.

Characteristic		Low expression of NPEPL1	High expression of NPEPL1
		*N* = 269	*N* = 270
Age, mean ± SD		61.09 ± 12.04	60.16 ± 12.15
Gender, *n* (%)	Female	91 (16.9%)	95 (17.6%)
	Male	178 (33%)	175 (32.5%)

Histologic grade, *n* (%)	G1	7 (1.3%)	7 (1.3%)
	G2	135 (25.4%)	100 (18.8%)
	G3	97 (18.3%)	110 (20.7%)
	G4	24 (4.5%)	51 (9.6%)

Pathologic stage, *n* (%)	Stage I	164 (30.6%)	108 (20.1%)
	Stage II	28 (5.2%)	31 (5.8%)
	Stage III	46 (8.6%)	77 (14.4%)
	Stage IV	30 (5.6%)	52 (9.7%)

T stage, *n* (%)	T1	166 (30.8%)	112 (20.8%)
	T2	34 (6.3%)	37 (6.9%)
	T3	66 (12.2%)	113 (21%)
	T4	3 (0.6%)	8 (1.5%)

N stage, *n* (%)	N0	126 (49%)	115 (44.7%)
	N1	6 (2.3%)	10 (3.9%)

M stage, *n* (%)	M0	236 (46.6%)	192 (37.9%)
	M1	28 (5.5%)	50 (9.9%)

**Table 2:** Univariate Cox regression analysis and multivariate Cox regression analysis of NPEPL1 and clinicopathologic parameters with OS in ccRCC.

Characteristics		Total (*N*)	Univariate analysis		Multivariate analysis	
			Hazard ratio (95% CI)	*P* value	Hazard ratio (95% CI)	*P* value
T stage *N* = 539	T1 and T2	349	3.228 (2.382–4.374)	**<0.001**	1.388 (0.610–3.158)	0.434
	T3 and T4	190				

N stage *N* = 257	N0	241	3.453 (1.832–6.508)	**<0.001**	1.258 (0.616–2.569)	0.529
	N1	16				

M stage *N* = 506	M0	428	4.389 (3.212–5.999)	**<0.001**	3.090 (1.804–5.291)	**<0.001**
	M1	78				

Gender *N* = 539	Female	186	0.930 (0.682–1.268)	0.648	NA	NA
	Male	353				

Age *N* = 539	≤60	269	1.765 (1.298–2.398)	**<0.001**	1.859 (1.211–2.852)	**0.005**
	>60	270				

NPEPL1 *N* = 539	Low	269	2.621 (1.900–3.615)	**<0.001**	2.401 (1.509–3.821)	**<0.001**
	High	270				

Pathologic stage *N* = 536	Stage I and stage II	331	3.946 (2.872–5.423)	**<0.001**	1.348 (0.532–3.415)	0.529
	Stage III and stage IV	205				
Histologic grade *N* = 531	G1 and G2	249	2.702 (1.918–3.807)	**<0.001**	1.508 (0.905–2.513)	0.115
	G3 and G4	282				

The indicators in bold are meaningful. Due to the limitation of prognostic statistics, we choose to retain 3 decimal places and use < 0.001 to represent meaningful indicators.

**Table 3:** The impact of high and low NPEPL1 expression for clinicopathologic parameters.

Characteristics	Total (*N*)	Odds ratio (OR)	*P* value
T stage (T3&T4 vs. T1&T2)	539	2.354 (1.640–3.398)	<0.001
N stage (N1 vs. N0)	257	1.826 (0.657–5.515)	0.258
M stage (M1 vs. M0)	506	2.195 (1.340–3.658)	0.002
Age (>60 vs. ≤60)	539	0.737 (0.525–1.034)	0.078
Gender (male vs. female)	539	0.942 (0.660–1.344)	0.741
Pathologic stage (stage III and stage IV vs. stage I and stage II)	536	2.345 (1.643–3.363)	<0.001
Histologic grade (G3&G4 vs. G1&G2)	531	1.766 (1.253–2.496)	0.001

## Data Availability

The datasets generated during and/or analyzed during the current study are available from the TGCA dataset and HPA dataset.
